# Microalgae-Derived Carotenoid Extract and Biomass Reduce Viability, Induce Oxidative Stress, and Modulate the Purinergic System in Two Melanoma Cell Lines

**DOI:** 10.3390/life15020199

**Published:** 2025-01-28

**Authors:** Luisa Chitolina Schetinger, Loren S. B. de Jesus, Nathieli B. Bottari, Altevir R. Viana, Jelson N. Nauderer, Marcylene V. Silveira, Milagros Castro, Pricila Nass, Patrícia Acosta Caetano, Vera Morsch, Eduardo Jacob-Lopes, Leila Queiroz Zepka, Maria Rosa Chitolina Schetinger

**Affiliations:** 1 Department of Food Science and Technology, Federal University of Santa Maria (UFSM), Santa Maria 97105-900, Brazil; luschetinger@gmail.com (L.C.S.); pricila.nass@gmail.com (P.N.); pati.caetano98@gmail.com (P.A.C.); ejacoblopes@gmail.com (E.J.-L.); 2 Department of Biochemistry and Molecular Biology, Federal University of Santa Maria (UFSM), Santa Maria 97105-900, Brazil; lorensborbaj@gmail.com (L.S.B.d.J.); nathieli_bb@hotmail.com (N.B.B.); rossato.viana@hotmail.com (A.R.V.); jelsonnauderer@gmail.com (J.N.N.); marcyvieirass@outlook.com (M.V.S.); mifave_11@hotmail.com (M.C.); veramorsch@gmail.com (V.M.); 3 Department of Microbiology and Parasitology, Biology Institute, Federal University of Pelotas (UFPEL), Pelotas 96010-610, Brazil

**Keywords:** carotenoids, adenosine, ATP, melanoma, purinergic system

## Abstract

Cutaneous melanoma (CM) is an aggressive and metastatic tumor, resulting in high mortality rates. Despite significant advances in therapeutics, the available treatments still require improvements. Thus, purinergic signaling emerged as a potential pathway to cancer therapy due to its involvement in cell communication, proliferation, differentiation, and apoptosis. In addition, due to safety and acceptable clinical tolerability, carotenoids from microalgae have been investigated as adjuvants in anti-melanoma therapy. Then, this work aimed to investigate the in vitro anti-melanogenic effect of carotenoid extract (CA) and total biomass (BM) of the *Scenedesmus obliquus* microalgae on two cutaneous melanoma cell lines (A375 and B16F10). Cells were cultivated under ideal conditions and treated with 10, 25, 50, and 100 μM of CA or BM for 24 h. The effects of the compounds on viability, oxidant status, and purinergic signaling were verified. The IC50 cell viability results showed that CA and BM decreased B16F10 viability at 24.29 μM and 74.85 μM, respectively and decreased A375 viability at 73.93 μM and 127.80 μM, respectively. Carotenoid treatment for 24 h in B16F10 and A375 cells increased the release of reactive oxygen species compared to the control. In addition, CA and BM isolated or combined with cisplatin chemotherapy (CIS) modulated the purinergic system in B16F10 and A375 cell lines through P2X7, A2AR, CD39, and 5′-nucleotidase. They led to cell apoptosis and immunoregulation by activating A2A receptors and CD73 inhibition. The results disclose that CA and BM from *Scenedesmus obliquus* exhibit an anti-melanogenic effect, inhibiting melanoma cell growth.

## 1. Introduction

Melanoma is the most lethal type of skin cancer that originates from the malignant transformation of melanocytes. Among the risk factors of malignant melanoma are UV radiation, family history, and fair skin, eyes, and hair [1]. Worldwide, the incidence of melanoma occupies around 1.7% of all newly diagnosed primary malignant cancers, and patients dying from melanoma account for 0.7% of all cancer mortality. The formation of metastasis is an integrative process with complexity and mainly accounts for the mortality of melanoma patients [2].

The development of multidrug resistance, multiple side effects, and the inflated costs of the commercial therapy existent for malignant melanoma are important grounds for the need to discover new compounds that are safe and more effective [3]. In recent years, there has been increasing interest in the therapeutic potential of purinergic signaling for cancer therapy. Purinergic signaling plays a key role in modulating inflammatory and immune responses by extracellular biomolecules such as adenine nucleotides (adenosine triphosphate [ATP], adenosine diphosphate [ADP], and adenosine monophosphate [AMP]) and their derived adenosine nucleoside [4]. Their effects depend on the concentration of nucleotides, the expression pattern of purinergic receptors and enzymes, and the general dynamics of their synthesis and degradation [5].

In the tumor microenvironment, cells release extracellular ATP (eATP) through pannexin channels, which can bind to P2X and P2Y receptors or can be hydrolyzed to ADP or AMP by ectonucleoside triphosphate diphosphohydrolase (also known as NTPDase or CD39) and converted into adenosine by 5′-nucleotidase enzymes (also known as ecto-5′-nucleotidase, 5-NT, or CD73) [6,7]. Depending on the P2 receptor subtypes expressed, tumor cells may be more sensitive to death-inducing factors or contribute to cancer progression. Thus, ATP-depleting strategies enhance anticancer agent activity. Furthermore, tumor progression was inhibited in CD73-deficient mice, while CD39 directly promoted tumor cell growth [8,9].

Moreover, P2X7 is one of the purinergic receptors that have attracted much attention during the last years in the context of cancer [10]. Once P2X7 is activated, the beneficial or detrimental role of ATP may depend on the mechanisms of cell death and pro-inflammatory and oxidant response involving the NLRP3 inflammasome, caspase-1, and the secretion of cytokines. Moreover, the activation of P2X7 causes a decrease in the A375 melanoma cell line by apoptosis, suggesting the inhibition of P2X7 can be a target of melanoma therapy [11].

ATP and adenosine (ADO) play crucial roles in the cancer environment. In the tumor microenvironment, the signaling involving these molecules and immune cells is regulated by an enzymatic chain and purinergic receptors, collectively known as the purinome. The A2A receptor (A2AR) particularly exhibits a pro-tumor function by diminishing the immune response and supporting malignant melanoma growth [12,13]. Thus, Gutknecht da da Silva et al. [14] investigated the impact of A2AR antagonism with Istradefylline (IST) on the purinergic signaling profile within melanoma tumors and immune compartments. They observed that IST treatment resulted in reduced melanoma tumor growth and inhibited the AKT/mTOR pathway, which is associated with tumor progression. In the tumor, spleen, and thymus, the modulation of purinergic enzymes (CD39, CD73, and E-ADA) indicated a pro-inflammatory profile, characterized by elevated extracellular ATP concentrations relative to ADO. Inhibiting A2AR led to a compensatory feedback mechanism, increasing A2AR expression at the tumor site. However, this also resulted in heightened expression of the P2X7 receptor (P2X7R), which amplified pro-inflammatory pathways, evidenced by the release of IL-1β and cytokines such as IFN-γ and TNF-α. Their findings highlight the interplay between A2AR and P2X7R expression and function. They suggested that IST holds potential as an off-label cancer treatment, promoting an anti-tumor response through the production of pro-inflammatory cytokines and inhibition of the AKT/mTOR tumor growth pathway [14,15,16,17].

More recent assays have pointed to microalgae as sources of new anticancer compounds, especially carotenoids [18]. Carotenoids are naturally colored liposoluble pigments present in plants, fungi, bacteria, and algae. Although many carotenoids are described in plants, microalgae have been particularly employed in the development of carotenoid-enriched products, such as food supplements or medicines [19]. In this novel scenario, microalgae have assumed an important role due to their easy cultivation, ability to adapt to cultural conditions on a large scale, and the possibility of production throughout the year, in addition to being considered renewable sources of carotenoids [20].

Likewise, carotenoids have been implicated in cancer prevention through their influence on pathways involved in cell growth and apoptosis. An in vitro study using B16 melanoma cells demonstrated that carotenoids exhibit significant anticancer activity [21]. Another study made significant strides in understanding the role of purinergic signaling and the anti-melanogenic effects of a natural compound in SK-MEL-28 melanoma cells. Caffeine was found to markedly decrease melanoma cell viability and migration while sparing non-tumor cells. It induced a rise in ROS levels and increased PSH levels within melanoma cells. Furthermore, caffeine downregulated the expression of CD39 and CD73, reduced the hydrolysis of ATP, ADP, and AMP nucleotides and elevated extracellular ATP levels. These findings highlight caffeine’s ability to reduce the viability and migration of metastatic cutaneous melanoma cells, enhance ROS production and boost PSH levels [22].

Research on natural extracts has identified promising discoveries. *Vassobia breviflora* has been investigated as a natural anti-melanogenic agent, with its modulatory capacity linked to the purinergic pathway. Notably, its compound N-methyl-(2S,4R)-*trans*-4-hydroxy-L-proline showed a higher binding affinity to both P2X7 and P2Y1 purinergic receptors, as indicated by receptor−ligand complex’s estimated binding affinity and evidenced by ∆G values. These results suggest a potential interaction of *V. breviflora* bioactive compounds with growth-inhibitory properties in B16F10 melanoma. This indicates that these compounds could be promising candidates for melanoma and cancer treatment [23].

Furthermore, among the various microalgae, *Scenedesmus obliquus* stands out as one of the most found genera in freshwater. Consequently, this species often becomes the focal point of numerous novel discoveries [24]. Notably, *Scenedesmus obliquus* contains plenty of bioactive compounds, including carotenoids, which have demonstrated the potential to regulate cellular activities, inhibit the proliferation of tumor cells and enhance the apoptosis of cancerous cells [25]. Particularly, the consumption of *Scenedesmus obliquus* resulted in a reduction of up to 70% in triglyceride levels in Wistar rats [26]. Furthermore, lutein extracted from *Scenedesmus obliquus* has been shown to enhance immune function and mitigate brain injury caused by cyclophosphamide in Wister rats [27]. Additionally, the ethanolic extract of *Scenedesmus obliquus* exhibited minimum inhibitory concentrations (MICs) of 2.25 mg/mL, 7.12 mg/mL, and 9.5 mg/mL against *E. coli*, *P. aeruginosa*, and *S. aureus*, respectively. GC-MS analysis revealed the presence of various bioactive compounds in the extract, contributing to its antibacterial properties [28]. Moreover, *Scenedesmus obliquus* has been applied in the synthesis of nanocompounds. For instance, a novel GaFe₂O₄@Ag nanocomposite was synthesized using an extract from *Scenedesmus obliquus* and evaluated for its anticancer properties against gastric cancer cells (AGS). The compound increased the expression of the BAX, Bcl-2, and CASP8 genes, indicating pro-apoptotic action [29].

The impact of carotenoids on B16F10 cells has been limitedly investigated in the last decades. Notably, the successful induction of apoptosis in lung metastatic melanoma (B16F10) was achieved through the utilization of an astaxanthin nanoemulsion derived from *Haematococcus pluvialis* [30]. Additionally, on B16F10 cells, fucoxanthin-triggered apoptosis was associated with the reduction in protein levels of Bcl-xL, an inhibitor of apoptosis proteins (IAPs). This led to a consecutive activation of caspase-9, caspase-3, and PARP [31]. Furthermore, the study of antitumor effects of carotenoids on A375 cells follows a similar pattern in the literature. For instance, melanoma cell line A375 was effectively restrained through the application of carotenoid extract and nanoemulsion derived from pomelo leaves [32]. Moreover, the cyanobacterium *Nostoc* sp. AARL C008 exhibited significant cytotoxicity against A375 malignant melanoma skin cancer cells, demonstrating an IC50 of 0.42 mg/mL [33].

Hence, additional research is needed to fully understand the influence of novel nutraceuticals, particularly those derived from microalgae and other natural sources, on cancer cells. This entails further exploration of the utilization of carotenoids in vitro models of A375 and B16F10, shedding light on apoptotic pathways and oxidative stress [34,35,36].

Thus, this present study aimed to investigate the possible antitumoral effects of carotenoids (CA) and total biomass (BM) in two melanoma cell lines (A375 and B16F10) through cell viability essays, oxidative stress, and the purinergic signaling pathway.

## 2. Materials and Methods

### 2.1. Preparation of Carotenoids Extract and Total Biomass of the Microalgae Scenedesmus obliquus

Axenic cultures of *Scenedesmus obliquus* (CPCC05) were obtained from the Canadian Psychological Culture Collection University of Toronto, Canada. Stock cultures were propagated on solidified agar (20 g/L) containing BG-11 synthetic medium. The *Scenedesmus obliquus* cultures were maintained at a photon flux density of 15 µmol photons m^−2^ s^−1^ at 30 °C, with an initial pH value of 8.0, a continuous aeration of 1 VVM (volume of air per volume of culture per minute) without CO_2_ enrichment, and a photoperiod of 12 h. BM production was performed in a hybrid photobioreactor operating in batch mode, fed with BG-11 synthetic medium [37].

The microalgae were separated from the culture medium by centrifugation at 1500× *g* and at 10 °C for 10 min (centrifuge model D-37520, Thermo, Langenselbold, Germany). The experiments with wet microalgae BM (95% humidity) were executed with the paste collected after centrifugation. For the experiments with dry biomass, the paste obtained after centrifugation was frozen at −18 °C for 24 h and lyophilized for 24 h at −50 °C under a pressure of 175 µm Hg. Samples were stored under refrigeration until analyses were made.

To determine the original CA content of *Scenedesmus obliquus* (control extract) and to obtain the isolated carotenoid extract for simulated digestion, the carotenoids were extracted according to the method of Rodrigues et al. developed in 2015 [38]. The pigments were extracted from the lyophilized biomass (0.1 ± 0.02 g) with a 90:36 (*v*:*v*) mixture of ethyl acetate and methanol, 10 mL, in a mortar and pestle, followed by centrifugation for 7 min at 1500× *g* (Thermo, Langenselbold, Germany).

Then, the extract was saponified (alkaline hydrolysis) for 16 h with 10 g/100 mL/1 KOH methanol at room temperature, and the alkali was removed with distilled water. The extract was concentrated on a rotary evaporator, placed in an N_2_ atmosphere and kept at −40 °C in the dark until analyses. Then, the micellarized pigments were extracted [34]. Lyophilized samples were obtained by adding 15 mL of a mixture of ethyl ether and petroleum ether (1:1) followed by 5 min ultrasonic cycles (see parameters in Section 2.2) and then centrifuged when the supernatant was collected. The process was repeated until the supernatant was colorless. After extracts were saponified and rotary evaporated, they were subjected to chromatography analyses. The complete phytochemical characterization of CA and BM can be accessed in previous work [39].

### 2.2. Carotenoid Extraction and Analysis

*Scenedesmus obliquus* carotenoids were extracted from aliquots of 0.1 ± 0.02 g from freeze-dried biomass with ethyl acetate and methanol using a mortar and pestle followed by centrifugation (Hitachi, Tokyo, Japan) (7 min; 1500× *g*). The homogenized sample suspension was filtered through a 0.22 µm polyethylene membrane and concentrated in a rotary evaporator (<30 °C); then, the extract was transferred to a mixture of petroleum ether/diethyl ether [1:1 (*v*/*v*)], and the extraction solvent was removed by washing [40,41]. Before HPLC-PDA analysis, the sample was solubilized in methanol (MeOH)/methyl tert-butyl ether (MTBE) (70:30) and filtered through Millipore membranes (0.22 µm). The mobile phases A (MeOH) and B (MTBE) used a linear gradient program as follows: from 0 to 30 min, 5% B; from 30 to 40 min, 5 to 30% B; from 40 to 41 min, 30 to 50% B; and from 41 to 50 min, 50 to 5% B. The flow rate was set at 0.9 mL/min, the injection volume was 20 µL, the column temperature was maintained at 22 °C, the UV/vis spectra were acquired between 220 and 700 nm, and the chromatograms were processed at 450 nm. Carotenoids were individually quantified by HPLC-PDA using six-point analytical curves (R^2^ = 0.99) of all-*trans*-lutein (1.0–50.0 and 0.05–10 µg/mL), all-*trans*-β-cryptoxanthin (1.0–60 µg/mL), and all-*trans*-β-carotene (1.0–50 and 0.05–10 µg/mL).

The MS/MS parameters were set according to Giuffrida et al. [42] with adaptations. The APCI interface operated in positive (+) mode; the other parameters were as follows: detector voltage: 4.5 kV; interface temperature: 350 °C; DL temperature: 250 °C; heat block temperature: 200 °C; nebulizing gas flow (N_2_): 3.0 L/min; drying gas flow (N_2_): 5.0 L/min; collision-induced dissociation (CID) gas: 23 kPa (argon); event time: 0.5 s. The identification quality was improved by using MS/MS simultaneously to select the ion monitoring and multiple reaction monitoring modes. Identification was performed according to the following combined information: elution order on the C30 HPLC column, co-chromatography with authentic standards, UV-Vis spectrum (spectral fine structure [λmax], the ratio of the height of the longest wavelength absorption peak [III] to the middle absorption peak [II], and the ratio of the cis peak [AB] to the middle absorption peak [II]), as well as mass characteristics protonated molecule ([M + H]^+^) and MS/MS (fragments), compared with data available in the literature [19,38,39,41,43,44].

### 2.3. Cell Culture and Treatment

Murine (B16F10) and human (A375) melanoma cell lines were used in this study to evaluate the antitumoral activity of *Scenedesmus obliquus* CA and BM. The cell lines were grown in Dulbecco’s Modified Eagle Medium (DMEM, Sigma-Aldrich, St. Louis, MO, USA), supplemented with 10% fetal bovine serum (Vitrocell) and 1% antibiotics (penicillin/streptomycin), as recommended by the Rio de Janeiro Cell Bank (BCRJ), in 25 cm^2^ polystyrene flasks (Corning). Cells were placed in an incubator with a humidified atmosphere and 5% CO_2_ at a temperature of 37 °C. The culture medium was replaced every 2–3 days, according to the cell line metabolism.

For cell expansion, first, cells were mechanically removed from the surface of the flask using a cell scraper. Then, cells in culture media were transferred to falcon tubes (Falcon tubes, Corning, Corning, NY, USA) and centrifuged for 5 min at 1500 rpm (Microcentrifuge tubes, Eppendorf, Hamburg, Germany). The cell pellet formed was resuspended in fresh culture medium and transferred to 96-well plates, at a concentration of 1 × 10^4^ cells per well. The cells were treated with 1, 10, 25, 50, and 100 µM of CA or BM to determine the concentration that inhibits 50% (IC50) of cell viability. For the dilution of BM, DMSO was first added to the biomass, followed by the culture medium, to achieve a final concentration of 0.1% before application to the cells [45].

The HFF-1 cell line (human fibroblast) was used as a cytotoxicity control for the treatment with CA and BM. Cells were cultivated in Dulbecco’s Modified Eagle Medium (DMEM, Sigma-Aldrich), supplemented with 10% fetal bovine serum (Cultilab, Campinas, SP, Brazil) and 1% antibiotics (penicillin/streptomycin), as recommended by the BCRJ. Cells were placed in an incubator with a humidified atmosphere and 5% CO_2_, at a temperature of 37 °C. HFF-1 cells were mechanically removed as previously described for cell expansion and centrifuged for 5 min at 1500 rpm. The cell pellet was resuspended in fresh culture medium and transferred to 96-well plates at a concentration of 1 × 10^4^ cells per well. Cells were treated with 1, 10, 50, and 100 µM of CA or BM. In some experiments, cisplatin was also used as a negative control and was applied in a concentration of 100 µM.

### 2.4. Evaluation of Cell Viability by the MTT Method

Cell viability was analyzed by the MTT method [46]. After the treatment period, 20 µL of a cell solution was added to wells previously containing the sterile MTT solution (5 mg/mL in 1 x PBS), and the plates were incubated for 4 h [47]. The medium was carefully removed, and the formazan crystals were dissolved by adding 200 µL of dimethylsulfoxide (DMSO). Cell growth inhibition was detected by measuring the absorbance at 570 nm in a plate reader (SpectraMax® i3x, Molecular Devices, San Jose, CA, USA). The results were expressed as the percentage of viable cells compared to the negative control.

### 2.5. Viability Detection by the Neutral Red Method

The neutral red method is based on the ability of viable cells’ lysosomes to uptake neutral red dye [47]. After treatment for 24 h, the culture medium was removed, and an incomplete medium containing neutral red dye at a concentration of 40 µg/mL was added for 5 h. Subsequently, the cells were washed to remove excess dye, and then, a solution containing 1% acetic acid, 50% ethanol, and 49% water was added to disrupt cellular structures. Neutral red uptake was measured at 540 nm in a plate reader (SpectraMax^®^ i3x—Molecular Devices). The results were expressed as a percentage of viable cells concerning the negative control.

### 2.6. Lactate Dehydrogenase Assay (LDH)

The lactate dehydrogenase colorimetric assay was performed according to the “CytoTox 96^®^ Non-Radioactive Cytotoxicity Assay” (Promega, Madison, WI, USA) Kit to assess the viability of the fibroblasts. After 24 h of treatment, 100 µL of the cell supernatant was collected and transferred to a new 96-well plate containing the reagent. After incubating for half an hour at room temperature and protection from light, the enzymatic reading was performed in a plate reader at a wavelength of 492nm. The results were expressed as a percentage of cytotoxicity.

### 2.7. Quantification of Double-Stranded Extracellular DNA Concentration (dsDNA PicoGreen^®^)

An additional assay was performed to quantify the concentration of double-stranded DNA (dsDNA) (or its absence) in the supernatant using the Quant-iT™ PicoGreen™ dsDNA Assay Kit, Thermo Fisher Scientific, Waltham, MA, USA. After the treatment period, the plates containing CA- and BM-treated cells were centrifuged, and 10 µL of the supernatant was transferred to a new culture plate (96-well plate, black). Then, 80 µL of 1x Tris-EDTA (TE) buffer (10 mmol/L Tris HCl and 1 mmol/L EDTA; pH = 7.5) and 5 µL of PicoGreen reagent were added. The plate was incubated for 5 min at room temperature (protected from light), and the reading was performed in a fluorimeter (SpectraMax^®^ i3x—Molecular Devices) at 520 nm of emission and 480 nm of excitation. Results were expressed as fluorescence values, which indicate DNA integrity.

### 2.8. Analysis of Oxidative Stress Markers

The quantification of reactive species was evaluated by measuring the presence of reactive species inside the cell, with the addition of dichlorofluorescein acetate (DCFH-DA) to the cell. Inside the cells, this molecule is deacetylated by intracellular esterase enzymes [48]. This reaction forms, in turn, a non-fluorescent product, 4,1-dihydrochlorofluorescein (DCFH). Cell suspension (10 µL) was incubated with 10 µL of DCFH for 1 h at room temperature. The results were expressed as fluorescence intensity in comparison to the control. Fluorescence was measured in a plate reader (SpectraMax^®^ i3x—Molecular Devices), with an excitation wavelength of 480 nm and an emission wavelength of 520 nm.

The content of nitrites, a stable metabolite of nitric oxide (NO), was determined by the Sun and Griess method [49]. The supernatant was incubated with an equal volume of Griess reagent, and the absorbance was measured at 540 nm using the SpectraMax® i3x Molecular Devices reader, Molecular Devices, San Jose, CA, USA. Total nitrite concentration was determined by optical density compared to negative control.

Lipid peroxidation was determined by the quantification of thiobarbituric acid reactive substances (TBARSs), using malondialdehyde as a standard curve, following the modified method of Jentzsch [50]. TBARS levels were expressed as nmol of malondialdehyde/mg of protein. The levels of protein thiols (PSHs) were determined according to Ellman [51] with adaptations and were expressed as a percentage of the control.

### 2.9. Nucleotides and Nucleoside Hydrolysis

The total volume of a cell extract (0.4–0.6 mg/mL protein) was added to the E-NTPDase or E-5′nucleotidase reaction mix and pre-incubated for 10 min at 37 °C to a final volume of 200 µL. The E-NTPDase activity was determined according to Lunkes [52]. The reaction was initiated by adding ATP or ADP as a substrate at a final concentration of 1 mM. The E-5′-nucleotidase activity was determined according to Heymann [53]. Phosphate released by the hydrolysis of ATP, ADP, and AMP was measured using KH_2_PO_4_ as a standard. Controls were prepared to correct for non-enzymatic hydrolysis, and all samples were analyzed in triplicate. The specific activities of the enzymes were expressed as nmol Pi released/min/mg of protein.

Adenosine deaminase (ADA) activity was determined according to [1,53,54] based on the direct measurement of ammonia produced when adenosine deaminase acts on excess adenosine. Briefly, 50 µL of cells reacted with 21 mmol/L of adenosine at a pH value of 6.5, and the mixture was incubated at 37 °C for 60 min. Subsequently, the reaction was stopped by adding a solution of 106.2 mM phenol, 67.8 mM sodium nitroprusside, and a solution of hypochlorite. The amount of ammonia produced was measured at 620 nm, and the results were expressed in units per liter (U/L).

### 2.10. Flow Cytometry Analysis of the Purinergic System

The expression levels of purinoreceptors (P2X7 and A2A) and adenosine deaminase enzyme (CD73) were measured by flow cytometry in B16F10 and A375 cells exposed to 50 µM of CA or BM. Cells were transferred to 5 mL polystyrene tubes (2 × 10^6^ cells/tube), and the medium was removed by centrifugation (400× *g*, 5 min). Cells were washed once with ice-cold PBS (600× *g*, 5 min centrifugation for solution removal) and then fixed and permeabilized with buffer containing 4% paraformaldehyde for 20 min on ice. After buffer removal (400× *g*, 5 min centrifugation), cells were washed twice with a solution containing 10% fetal bovine serum in DMEM medium and centrifuged at 400× *g* for 5 min. Then, cells were resuspended in the same solution for antibody labeling using anti-P2X7 (1:500, Santa Cruz Biotechnology, Dallas, TX, USA), anti-A2A (1:500, Santa Cruz), and anti-CD73 (1:500, Santa Cruz). Following a washing step with PBS, cells were incubated with Alexa Fluor 488 (1:500; Life Technologies, Carlsbad, CA, USA) and analyzed by flow cytometry (FACS-VERSE Cytometer, BD Biosciences, San Jose, CA, USA). Thirty-thousand events were acquired per sample. Forward and side light-scatter signals were used to exclude dead cells and debris. Data were analyzed using the FlowJo V10 software (FlowJo, Ashland, OR, USA).

### 2.11. Statistical Analysis

Data analyses were performed using the GraphPad Prism software V7.0 (GraphPad Prism, San Diego, CA, USA). One-way and two-way analysis of variance (ANOVA) were performed to compare treatments, followed by Tukey’s post hoc test. Statistically significant values were considered as follows: * *p* < 0.05; ** *p* < 0.01; and *** *p* < 0.001.

## 3. Results

### 3.1. HPLC-PDA-MS Characterization of Carotenoids

After analysis in HPLC-PDA-MS, the extract revealed the presence of all-trans-neoxanthin, 9-*cis*-neoxanthin, 15-*cis*-lutein, 13-*cis*-lutein, all-trans-lutein, all-trans-zeaxanthin, 2′-dehydrodeoxymyxol, all-trans-echinenone, 9-*cis*-echinenone, all-trans-α-carotene, all-trans-β-carotene, and 9-*cis*-β-carotene (Table 1). The compounds were quantified, and the results revealed an abundance of all-trans-β-carotene, all-trans-zeaxanthin, all-trans-lutein, 2′-dehydrodeoxymyxol, all-trans-echinenone, 9-*cis*-echinenone, all-trans-neoxanthin, 9-*cis*-neoxanthin, 9-*cis*-β-carotene, 15-*cis*-lutein, all-trans-α-carotene, and 13-*cis*-lutein in Table 1 and Table 2.

### 3.2. Carotenoids and Biomass Reduce Melanoma Cell Viability

**Figure 1:**
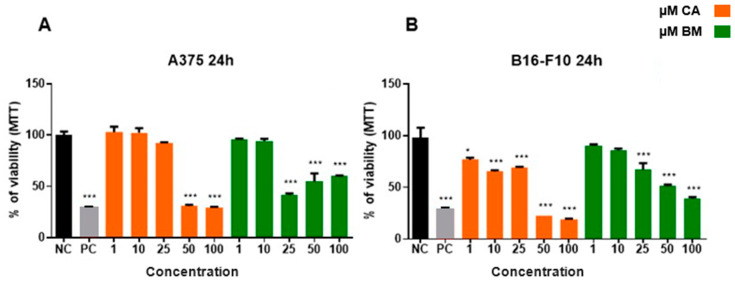
Cell viability of A375 (**A**) and B16F10 (**B**) cells exposed to a range of concentrations of carotenoid and total biomass in 24 h of treatment, measured by the MTT assay. Statistical analysis compared treatment groups to the negative control (NC) and to the positive control (PC). Statistically significant values were considered as follows: * *p* ≤ 0.05; *** *p* ≤ 0.001.

The effects of CA and BM on A375 and B16F10 cell viability were evaluated using the MTT method (Figure 1). The data reveals that treatment of A375 cells (Figure 1A) with CA concentrations of 50 and 100 µM decreased cell viability by 70% and 77%, respectively, when compared to the negative control. Treatment of A375 cells with BM concentrations of 25, 50, and 100 µM decreased cell viability by 32%, 48%, and 60.2%, respectively, when compared to the negative control.

In Figure 1B, it is notable that B16F10 cells treated with CA concentrations of 50 and 100 µM decreased cell viability by 69.5% and 71%, respectively, compared to the negative control. In addition, B16F10 cells exposed to BM concentrations of 25, 50, and 100 µM decreased cell viability by 58.5%, 45.4%, and 40%, respectively, compared to the negative control.

### 3.3. Effects of Different Concentrations of Carotenoids and Biomass in a Fibroblast Cell Line (HFF-1)

**Figure 2:**
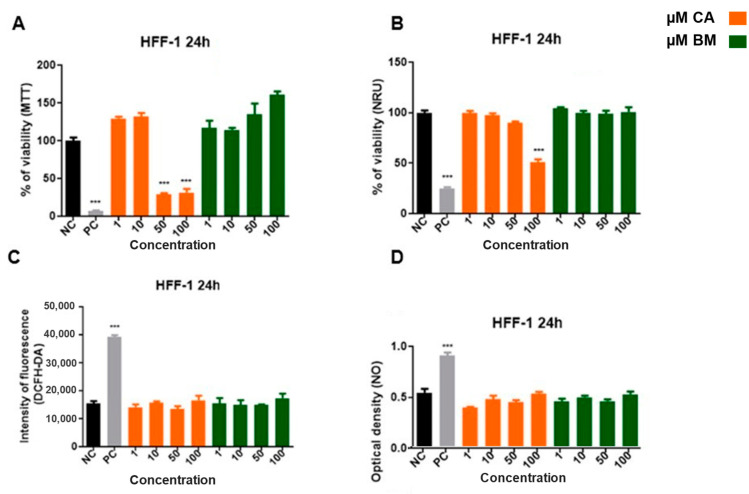
Cell viability and oxidative stress quantification after treatment with different concentrations of carotenoids and total biomass: (**A**) MTT method; (**B**) neutral red method; (**C**) dichlorofluorescein method; (**D**) nitric oxide release measurement. Statistical analyses were performed comparing treatment and control groups (NC). Statistically significant values were considered as follows; *** *p* ≤ 0.001. NC—negative control (cells + culture medium); PC—positive control (100 mM H_2_O_2_).

To verify whether the cytotoxic concentrations of CA and BM in melanoma cells would cause a viability reduction and cytotoxicity on normal cells, the fibroblast cell line (HFF-1) was assessed. CA and BM effects on HFF-1 cell viability are shown in Figure 2. The concentration of 50 and 100 µM of CA significantly reduced the percentage of viable cells compared to the negative control (Figure 2A). Likewise, 100 µM of CA decreased neutral red cell uptake viability (*p* < 0.0001) (Figure 2B). No differences were observed in ROS and NO analyses (Figure 2C,D) (*p* > 0.05).

### 3.4. Cytotoxic Effects of Carotenoids and Biomass on Melanoma Cells

**Figure 3:**
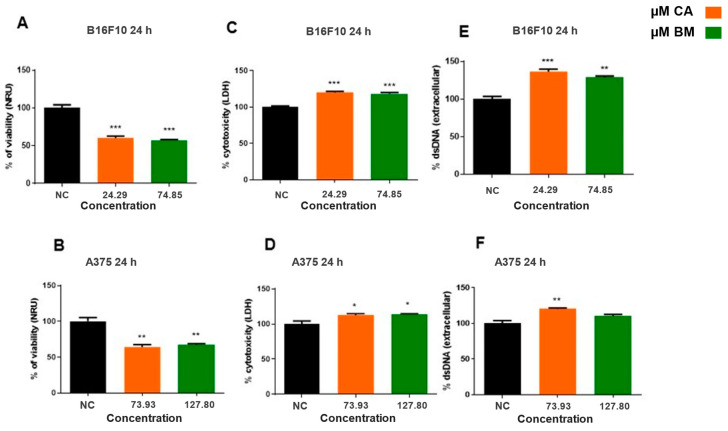
Determination of cytotoxicity by MTT (**A**,**B**), LDH (**C**,**D**), and free dsDNA (**E**,**F**) in B16F10 and A375 cells exposed to IC50 values for BM and CA. Statistical analysis was performed comparing treatment groups to negative control (NC), and statistically significant values were considered as follows: * *p* ≤ 0.05; ** *p* ≤ 0.01; *** *p* ≤ 0.001. Tukey’s post hoc test; two-way ANOVA.

Determination of cytotoxicity by MTT, LDH, and free dsDNA is presented for B16F10 and A375 cell lines utilizing the IC50 values (Figure 3). IC50 values obtained were 24.29 µM for CA and 74.85 µM for BM in the B16F10 cell line, and the IC50 values of 73.93 µM for CA and 127.80 µM for BM were identified in the A375 cell line. The results reveal a cytotoxic effect of CA and BM in melanoma cell lines (Figure 3A,D). Both extracts have shown cytotoxic effects, as evidence by reduced the percentage of cell viability, augmented lactate dehydrogenase levels, and dsDNA compared to the negative control in both A375 (Figure 3B,D,F) and B16F10 (Figure 3A,C,E) lineages. The MTT and LDH cell viability analyses were corroborated by the quantification of free dsDNA (Figure 3C,F). The PicoGreen^®^ assay confirmed the changes in mitochondrial cell viability, showing a concentration-dependent behavior related to the incubation time. The release of dsDNA in the supernatant occurred at the IC50 values.

### 3.5. Carotenoids and Biomass Reduce Colony Formation in Melanoma Cells

**Figure 4:**
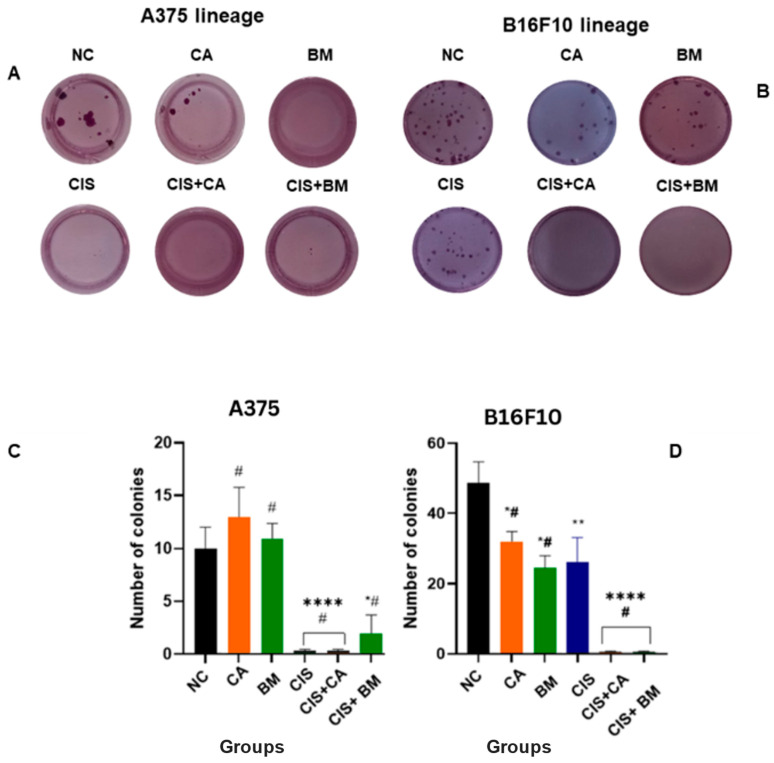
Effects of carotenoids and biomass in colony formation. Treatment concentrations applied: 50 µM of CA or BM, 100 µM of cisplatin, or a combination of them. A negative control (NC) was also present: (**A**,**C**) A375 lineage; (**B**,**D**) B16F10 lineage. Statistical analysis was performed comparing treatment groups to the negative control (NC), and statistically significant values were considered as follows: * *p* ≤ 0.05; ** *p* ≤ 0.01; **** compared to negative control; # difference among treatment groups. the Tukey’s post hoc test; two-way ANOVA.

After determining the minimum concentrations of CA and BM capable of inhibiting 50% (IC50) of cell viability in human and murine melanoma cell lines, these effects were compared to those of cisplatin, a widely used chemotherapeutic agent [55,56]. As shown, the extracts alone exhibited cytotoxic and oxidant activity in melanoma cells while remaining safe for normal cells (HFF-1). Co-treatment with cisplatin-based chemotherapy (representative images are shown in Figure 4) significantly reduced the number of colonies in both A375 and B-16F10 cell lines compared to the negative control (*p* < 0.001). The combinations of CIS + CA and CIS + BM also reduced the number of colonies in A375 and B16F10 cells. While CA alone increased the number of colonies, BM alone decreased the number of colonies in both A375 and B16F10 cells (*p* < 0.05).

### 3.6. Oxidative Stress in A375 and B16F10

**Figure 5:**
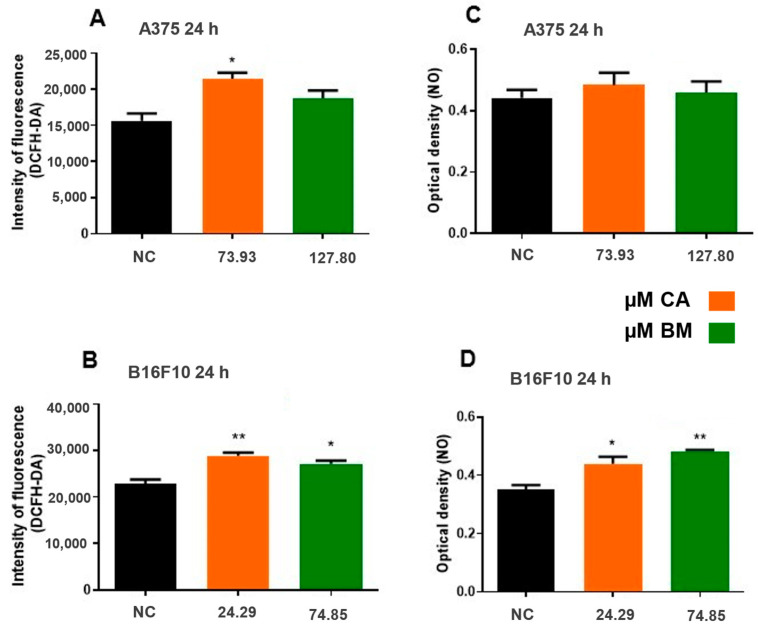
Assessment of the redox status of A375 and B16F10 cells exposed to a range of carotenoid concentrations and total biomass treatments. Results are presented for DCFH-DA (**A**,**B**) and NO (**C**,**D**). Statistical analysis was performed com-paring treatment to control groups (NC), and statistically significant values were considered as follows: * *p* ≤ 0.05; ** *p* ≤ 0.01.

The oxidant status in A375 and B16F10 cell lines exposed to CA and BM was assessed using a fluorescence assay (DCFH-DA) and measurements of nitric oxide (NO) levels. Cells exposed to CA showed a significant increase in the release of reactive species compared to the control group in both cell lines (*p* < 0.01). In B16F10 cells, BM exposure led to increased fluorescence intensity 24 h post-exposure compared to the negative control. Additionally, nitric oxide release was stimulated in B16F10 cells exposed to CA and BM compared to the negative control. However, in the A375 cell line, the compounds did not alter nitric oxide release (Figure 5).

### 3.7. Carotenoids and Biomass Modulate Nucleotides and Nucleoside Hydrolysis in Melanoma Cells

**Figure 6:**
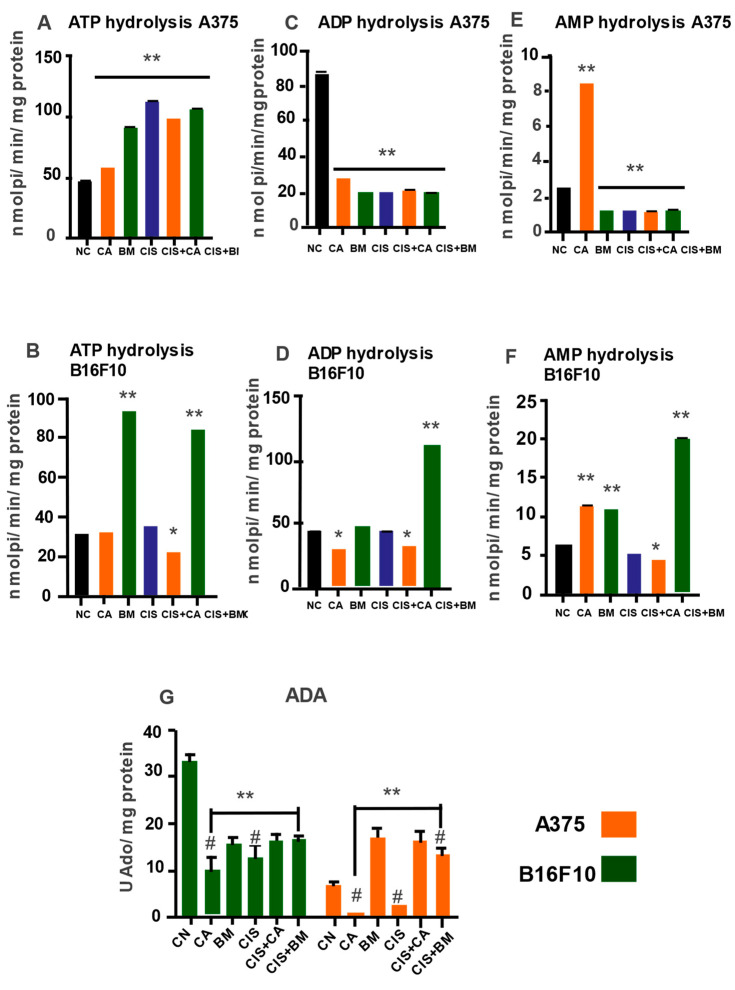
Hydrolysis of extracellular nucleotides and nucleosides in melanoma cells exposed to carotenoid extracts and total biomass: (**A**,**B**) NTPDase1 (using ATP as a substrate); (**C**,**D**) NTPDase1 (using ADP as a substrate); (**E**,**F**) 5′-Nucleotidase; (**G**) Adenosine deaminase (E-ADA). The treatment concentration applied were 50 µM for both CA and BM. Cisplatin was used at a concentration of 100 µM. Then, combinations of these same concentrations were performed. Values are expressed as mean ± SEM. Data were analyzed by two-way ANOVA followed by Tukey’s post hoc test. A statistical analysis compared treatment and control groups (NC). Statistically significant values were considered as follows: * *p* ≤ 0.05; ** *p* ≤ 0.01; # difference among treatment groups.

The hydrolysis of ATP, ADP, AMP, and adenosine was measured by NTPDase, 5′-NT, and ADA enzymes, as shown in Figure 6. CA, BM, and CIS, as well as CIS + CA and CIS + BM, increased NTPDase activity when ATP was used as a substrate compared to control in the A375 cell line (Figure 6A). In B16F10, BM and CIS + BM elevated ATP hydrolysis compared to the negative control (Figure 6B). The NTPDase-1 activity using ADP as a substrate was reduced by carotenoid and biomass extracts in the A375 cell line (Figure 6C) when compared to the negative control. The same effect was observed when cisplatin was added alone or in a combination with the extracts (*p* < 0.05). In murine melanoma (B16F10; Figure 6D), the carotenoid extract combined with cisplatin subtly decreased ADP hydrolysis compared to the control group (Figure 6D).

Furthermore, it was observed that, in A375 and B16F10 cells exposed to carotenoid extract, the hydrolysis of isolated AMP nucleotide increased, although the enzymatic activity was reduced when combined with cisplatin (Figure 6E). On the other hand, total biomass alone or combined with cisplatin significantly reduced AMP hydrolysis in the A375 cell line (Figure 6) when compared to the control. The opposite effect was observed in the B16F10 cell line (Figure 6F).

The deamination of the final product of AMP hydrolysis was evaluated by the activity of the enzyme adenosine deaminase (Figure 6G). Treatment of CA and BM in B16F10 cells reduced ADA activity when compared to the control group. The same effect was observed when combined with cisplatin. On the other hand, in A375 cells, the extracts showed antagonistic effects and the carotenoid decreased ADA activity, while the biomass increased ADA activity, compared to the negative control. Nevertheless, the association of the extracts with cisplatin promoted an increase in ADA activity in A375 cells when compared to the control group (Figure 6G).

### 3.8. Expression of Purinoreceptors Mediated by Carotenoids and Biomass in Melanoma Cells

**Figure 7:**
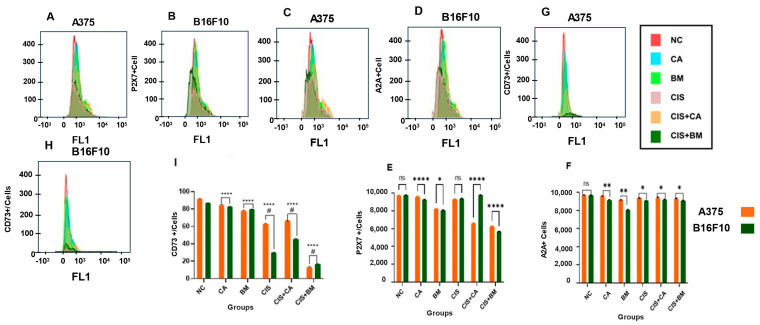
Carotenoids and biomass upregulate purinoreceptors in melanoma cells: (**A**–**D**) representative flow cytometry histograms; (**E**) expression of P2X7+ cells; (**F**) expression of A2A+ cells; (**G**,**H**) representative flow cytometry histograms; (**I**) expression of CD73+ cells. Data acquisition was performed by flow cytometry. The treatment concentrations applied were 50 µM for both CA and BM. Cisplatin was used at a concentration of 100 µM. Then, combinations of these same concentrations were performed. Values are expressed as mean ± SEM. Data was analyzed by two-way ANOVA followed by Tukey’s post hoc test. A statistical analysis was performed to compare treatment and control groups (NC). Statistically significant values were considered as follows: * *p* ≤ 0.05; ** *p* ≤ 0.01; **** compared to negative control; # difference among treatment groups.

In addition to ectonucleotidases activity, purine receptors P2X7, A2A, and CD73 enzymes were analyzed by flow cytometry (Figure 7). Data have shown a significant decrease in the expression of the P2X7 and A2A receptors in A375 and B16F10 cells after treatment with CA, BM, and the combinations of CIS + CA and CIS + BM, compared to the negative control (Figure 7E,F).

CD73-positive cells were shown diminished with CA, BM, CIS, and the combinations of CIS + CA and CIS + BM treatments in A375 and B16F10 melanoma cell lines when compared to the negative control group (Figure 7I).

## 4. Discussion

Microalgae are being successfully utilized in the food and pharmaceutical industries, with several studies highlighting their potential in cancer research due to their rich sources of various compounds, such as lutein, β-carotene, and zeaxanthin [57]. Thus, this study examined the carotenoid profile extracted from *Scenedesmus obliquus* and their potential effects on cell viability and oxidative stress parameters in A375 and B16F10 melanoma cell lines. Additionally, the study investigated the purinergic markers associated with carotenoids and biomass in melanoma cell lines A375 and B16F10.

The HPLC-PDA-MS analysis of the carotenoid extract from *Scenedesmus obliquus* revealed the presence of twelve relevant carotenoid compounds out of which all-*trans*-β-carotene, all-*trans*-zeaxanthin, all-*trans*-lutein, all-*trans*-echinenone, 9-*cis*-echinenone, 2′-dehydrodeoxymyxol, all-*trans*-neoxanthin, 9-*cis*-neoxanthin, and 9-*cis*-β-carotene were present in larger quantities. The activity of some of these compounds against various types of cancer cells has been reported: β-carotene [58,59,60,61]; zeaxanthin [62,63,64]; lutein [65,66,67]; neoxanthin [68,69,70]; and echinenone [71,72]. Thus, the activity of the *Scenedesmus obliquus* carotenoid extract observed in this experiment may be attributed to various compounds that could act individually or synergistically against A375 and B16F10 melanoma cells.

This study explored the potential antitumor activity of carotenoids and total biomass derived from the microalgae *Scenedesmus obliquus* against two melanoma cell lines, comparing their effects to those of the chemotherapeutic agent cisplatin. As anticipated, cisplatin treatment demonstrated a cytotoxic effect on the B16F10 and A375 cell lines after 24 h. These findings align with those of Dasari et al. [73], which indicated that cisplatin induces DNA damage and generates reactive oxygen species (ROS) within treated cells.

Likewise, treatments with carotenoids at concentrations of 50 µM and 100 µM for 24 h were able to reduce the cell viability of B1610 and A375 cells. Carotenoids are a class of pigments widely distributed in nature and present daily in human food. In addition to their coloring properties, these compounds exhibit significant biological activities, including antioxidant effects through the capture and neutralization of free radicals, as well as the induction of apoptosis via the modulation of various apoptotic molecules and pathways [74]. Concerning melanoma, some carotenoids have shown significant cytotoxic, antiproliferative, pro-apoptotic, and anticancer effects in in vitro and/or in vivo models [20,21].

In this study, CA and BM reduced the viability of B16F10 and A375 melanoma cell lines at higher concentrations (50 and 100 µM), revealing an anti-melanogenic effect. These results are evidenced by colony count, which was reduced in carotenoids and biomass treatment of melanoma cells. These findings are consistent with several studies demonstrating that carotenoids exhibit a cytotoxic effect comparable to that of drugs currently used in the treatment of metastatic melanoma [75,76,77].

The cytotoxicity of carotenoids and biomass was also evaluated using MTT and lactate dehydrogenase levels, which revealed the cytotoxic potential of CA and BM extracts at concentrations equivalent to 73.96 µM and 127.80 µM, respectively, in the A375 human melanoma cell line. In the B16F10 murine cell line, the minimum inhibitory concentrations of CA and BM extracts were 24.29 µM and 74.85 µM, respectively. In a study conducted by Juan-García et al. [78], the authors described the IC50 of carotenoids isolated from *Lycium barbarum* in the Caco-2 cell line as 12.5 µM after 24 h of treatment. Similar results were obtained in U-937 and HL-60 cell lines [77], evaluated by MTT and neutral red (NR) assay within 24 h of treatment. Although IC50 values vary among studies, due to isolation and extraction methods and phytochemical composition processes, they were in the same range as those reported here.

Two carotenoids (Bixin and norbixin) isolated from *Bixa orellana* seeds showed marked cell morphology change, nuclear condensation, DNA fragmentation, and mitochondrial dysfunction by the depletion of glutathione activity, as well as induced ROS generation [21]. These effects were also observed 24 h after the CA and BM exposition in the present study.

The functional modifications described in this paper are related to the structure of carotenoids, mainly due to their long system of conjugated double bonds [79,80]. Chemically, they are presented as the basic structure of a tetraterpene, with 40 carbon atoms formed by eight isoprenoid units of five carbons, connected in a linear arrangement with inverted symmetry in the center. Like this, carotenoids can act in the deactivation of reactive species, thus preventing the initiation of oxidation chains at the cellular level that leads to damage to deoxyribonucleic acid (DNA) and lipid peroxidation.

It is known that during inflammatory processes, such as in melanoma formation and growth, there is a release of reactive oxygen species (ROS), including superoxide anion, hydrogen peroxide (H_2_O_2_), and hydroxyl radicals (OH), which are produced not only by specialized phagocytic cells but also during normal oxidative metabolism and proliferative and apoptotic processes. Here, a pro-oxidant effect of carotenoids and total biomass was observed, assessed through the fluorescence intensity of DPPH and the equivalent optical density of nitric oxide release, using IC50 values.

Moreover, the carotenoid treatment for 24 h in B16F10 and A375 cells increased the release of reactive oxygen species when compared to the control. This effect could be mediated by the interference of carotenoids in the lipid peroxidation cascade. Several studies have described the potential of β-carotene as a radical scavenger against reactive species, namely ONOO− (peroxynitrite), and H_2_O_2_ in non-cellular and cellular systems by [81].

The presence of carotenoids in the medium leads to the formation of large carotene-radical complexes due to the oxidative effects of these compounds on subcellular organelles, such as free mitochondria. This may explain the observed dsDNA release. Additionally, high levels of reactive oxygen species (ROS) are known to function as second messengers in signal transduction pathways involved in tumor cell growth, transformation, and apoptosis [82].

To elucidate the possible underlying mechanism through which CA and BM extracts affect melanoma cells, the purinergic signaling pathway was evaluated, and the results of the hydrolysis of nucleotides and extracellular nucleosides were presented. The tumor microenvironment is rich in extracellular ATP, and its effect depends on both the concentration of ATP and the rate of degradation to adenosine by ectonucleotidases [3]. The results in this study point to an increase in the activity of the NTPDase enzyme when the CA and BM extract isolated and combined with CIS was incubated with ATP in A375 cells; the same phenomenon was observed with the BM and CIS + BM groups of treatments in B16F10 melanoma cells, suggesting an increase in extracellular ATP in the medium of melanoma cells. Previous work has shown that, even after surgical excision, melanoma patients have increased levels of extracellular ATP. In this case, ATP signaling increases the interaction between tumor cells and immune cells, causing an immune response [4,83].

Furthermore, carotenoid-induced apoptosis in melanoma cells may lead to the release of higher concentrations of ATP, which is subsequently hydrolyzed by NTPDases. Elevated levels of extracellular ATP can act as a danger signal, triggering an innate immune response. This process involves the activation of P2 and P1 transmembrane receptors, prompting macrophages to release a range of pro-inflammatory cytokines [84,85].

Carotenoids decreased NTPDase activity when ADP was used as a substrate in the A375 cell line. These results can be associated with the modulation of NTPDase by carotenoids. While immunosuppression caused by differences in ATP and adenosine levels acts on tumor progression, pharmacological modulation of nucleotide ADP, AMP, and nucleoside adenosine degradation enzymes in the tumor microenvironment may provide efficient means to reverse this condition [86,87].

At the same time, carotenoids exert an immunomodulatory effect via adenosine. Increased ADA activity suggests that high levels of adenosine are released. As adenosine is an anti-inflammatory molecule, it can be presumed that these molecules mediate an immunosuppressive response to protect adjacent tissues from inflammation and tumorigenesis. This nucleoside has been reported to be anti-proliferative and anti-angiogenic and to act as an immunosuppressive agent [11].

Some reports on P2 receptors in cancer cells indicate that high concentrations of ATP could enhance cancer growth and contribute to malignancy. This occurs mainly by activating P2X7, a receptor involved in cell proliferation, whose expression has been observed to increase in human melanoma [86,87]. The expression of P2X7 expression in A375 and B16F10 treated with CA, BM, and the combinations of CIS + CA and CIS + BM increased compared to the control. Possibly, CA and BM could act as agonists of the P2X7 receptor which may lead to apoptosis. Therefore, this study shows that alternative treatment with carotenoids and biomass promotes anti-melanoma responses, probably leading to decreased tumor growth and diminished invasiveness of malignancies by P2X7 modulation [88,89].

In the context of purinergic signaling, recent studies have shown that a single blockade of the A2A receptor, or in combination with PD-L1 or CTLA4 inhibitors, induces a defined T-cell activation, which limits tumor growth, leads to a Th1 gene expression signature consistent with immune activation and reinforces the memory immune cells [90]. Here, the mechanisms contributing to the immune escape of melanoma are also investigated by the A2A and CD73. The combination of cisplatin with treatments (CIS + CA and CIS + BM) increased A2A receptors and decreased CD73-positive cells, suggesting an inhibitor effect of the compounds, reinforcing the hypothesis that restraining the adenosine pathway by blocking the A2AR signaling pathway or reducing its production may increase the activity of anti-CTLA4 inhibitors and apoptosis [91,92].

In summary, the inhibition of the CD73 enzyme increased extracellular adenosine, which possibly acts as a negative immune checkpoint molecule that plays a role in the establishment of immune modulation in the melanoma microenvironment by ligation of the A2A receptor. These results indicate a new mechanism by which CA and BM could exert their protective roles in melanoma and support the idea that purinoreceptors-mediated signaling may be a crucial therapeutic target in the treatment of melanoma.

Thus, the results demonstrate that combining BM with cisplatin represents the most effective and safest approach against melanoma. This combination not only enhances antiproliferative effects but also modulates key enzymes and purinergic receptors involved in tumor progression. While CA exhibited higher cytotoxicity compared to BM when used alone, the observed synergy of BM with cisplatin highlights its potential as a promising strategy, effectively balancing potent antiproliferative activity with safety in normal cells.

## 5. Conclusions

In conclusion, this study demonstrates that carotenoids and total biomass extracted from the microalgae *Scenedesmus obliquus* exhibit significant anti-melanogenic effects on melanoma cell lines B16F10 and A375, with CA requiring lower concentrations than the biomass to achieve comparable effects. However, combining BM with cisplatin represented the most effective and safest approach against melanoma. Both the extract and the biomass reduced melanoma cell viability and possibly induced pro-apoptotic and immunomodulatory effects, mediated by the P2X7 and A2A receptors. These findings highlight the crucial role of these receptors in melanoma cell regulation and suggest their potential as therapeutic targets. Nonetheless, it is important to emphasize that this study was conducted in vitro. Therefore, further in vivo studies and clinical investigations are essential to confirm the efficacy and safety of these natural compounds for potential applications in melanoma therapy. This research lays a foundational for the development of adjuvant therapies utilizing carotenoids and microalgal biomass and underscores the necessity of further exploration for future biomedical advancements.

## Figures and Tables

**Table 1:** Chromatographic, UV-Vis spectrum, mass characteristics of *Scenedesmus obliquus* carotenoids obtained by HPLC-PDA-MS/MS.

Peak	Carotenoids	t_R_ (min) ^a^	UV-Vis Characteristics				Fragment Ions (Positive Mode) (*m*/*z*)
			λ_máx_ (nm) ^b^	III/II (%) ^c^	A_B_/II (%) ^d^	[M + H]^+^	MS/MS
1	all-*trans*-neoxanthin	7.4	415, 438, 468	78	0	601	583 [M + H − 18]^+^, 565 [M + H − 18 − 18]^+^, 547 [M + H − 18 − 18 − 18]^+^, 509 [M + H − 92]^+^
2	9-*cis*-neoxanthin	7.8	325, 415, 438, 467	81	16	601	583 [M + H − 18]^+^, 565 [M + H − 18 − 18]^+^, 547 [M + H − 18 − 18 − 18]^+^, 509 [M + H − 92]^+^
3	15-*cis*-lutein	10.0	331, 415, 437, 465	37	31	569	551 [M + H − 18]^+^, 533 [M + H − 18 − 18]^+^, 495, 477 [M + H − 92]^+^, 459
4	13-*cis*-lutein	10.9	328, 415, 438, 465	25	39	569	551 [M + H − 18]^+^, 533 [M + H − 18 − 18]^+^, 495, 477 [M + H − 92]^+^, 459
5	all-*trans*-lutein	11.9	422, 443, 471	57	0	569	551 [M + H − 18]^+^, 533 [M + H − 18 − 18]^+^, 495, 477 [M + H − 92]^+^, 459
6	all-*trans*-zeaxanthin	14.0	425, 449, 475	25	0	569	551 [M + H − 18]^+^, 533 [M + H − 18 − 18]^+^, 495, 477 [M + H − 92]^+^, 459
7	2′-dehydrodeoxymyxol	19.4	445, 474, 504	63	0	567	549 [M + H − 18]^+^
8	all-*trans*-echinenone	23.2	462	nc ^e^	0	551	533 [M + H − 18]^+^, 427, 203
9	9-*cis*-echinenone	25.4	342, 450	nc	20	551	533 [M + H − 18]^+^, 427, 203
10	all-*trans*-α-carotene	27.6	420, 445, 473	62	0	537	444 [M + H − 92]^+^, 399, 355
11	all-*trans*-β-carotene	31.5	425, 451, 476	25	0	537	444 [M + H − 92]^+^, 399, 355
12	9-*cis*-β-carotene	33.5	341, 420, 446, 472	20	14	537	444 [M + H − 92]^+^, 399, 355

^a^ t_R_—retention time on the C30 column; ^b^ linear gradient of MEOH:MTBE; ^c^ spectral fine structure: ratio of the height of the longest wavelength absorption peak (III) to that of the middle absorption peak (II); ^d^ ratio of the *cis* peak (A_B_) to the middle absorption peak (II); ^e^ nc—not calculated.

**Table 2:** HPLC-PDA quantification of carotenoid extract from *Scenedesmus obliquus* (concentration in (µg/g) of carotenoid extract).

Peak	Carotenoids	Concentration ^a^ (µg/g)
1	all-*trans*-neoxanthin	44.05 ± 2.98
2	9-*cis*-neoxanthin	41.34 ± 1.23
3	15-*cis*-lutein	8.86 ± 0.52
4	13-*cis*-lutein	7.27 ± 1.01
5	all-*trans*-lutein	64.76 ± 4.87
6	all-*trans*-zeaxanthin	67.92 ± 3.90
7	2′-dehydrodeoxymyxol	54.15 ± 1.76
8	all-*trans*-echinenone	50.36 ± 4.10
9	9-*cis*-echinenone	46.58 ± 1.89
10	all-*trans*-α-carotene	8.78 ± 1.05
11	all-*trans*-β-carotene	78.58 ± 4.08
12	9-*cis*-β-carotene	38.94 ± 2.57

^a^ Values are presented as mean ± standard error of the mean (SEM) from three independent experiments.

## Data Availability

The data presented in this study are available on request from the corresponding author.
